# Isolated Spherophakia and Glaucoma

**DOI:** 10.1155/2013/516490

**Published:** 2013-09-11

**Authors:** Joseph Pikkel, Epstein Irena

**Affiliations:** ^1^Ziv Medical Center, 13100 Safed, Israel; ^2^Faculty of Medicine, Bar Ilan University, 13150 Safed, Israel

## Abstract

We report a case of spherophakia that caused glaucoma and describe the characteristics and the ultrasound biomicroscopy findings as well as the mechanism and management of glaucoma in spherophakia. We suggest considering lens extraction to manage glaucoma in spherophakia and discuss the surgical considerations and possible complications of such an intervention.

## 1. Introduction

Spherophakia is a rare condition in which the crystalline lens assumes a spherical shape with an increased anterior-posterior diameter and a reduced equatorial diameter. Spherophakia can occur as an isolated anomaly or associated with a systemic disorder such as the Weill-Marchesani syndrome, Marfan's disorder, mandibulofacial dysostosis, Alport's syndrome, and Klinefelter's syndrome [1–3]. 

We report a case of spherophakia that caused glaucoma. We describe the characteristics and the ultrasound biomicroscopy findings as well as the mechanism and management of glaucoma in spherophakia. 

The role of lens extraction in the management of glaucoma in spherophakia has not been established; we discuss the surgical considerations and possible complications of such an intervention. 

## 2. Patient Description

A 72-year old man was referred to our outpatient clinic suspected of suffering from uveitis in his left eye. He initially presented 2 years ago with acute angle-closure glaucoma in his right eye that was treated with lowering the intra ocular pressure by local beta blockers and local prostaglandins followed by neodymium: YAG laser peripheral iridotomies in both eyes. Intraocular pressure values were 36 mmHg in the right eye and 18 mmHg in the left eye on admission and lowered to 16 mmHg in the right eye and 12 mmHg in his left eye before performing the iridotomies. After that, the IOP was within normal limits for 2 years, and there is no documentation of recurrent iritis in either eye in this period of time. 

The patient had chronic renal failure, cardiac arrhythmia, and arterial hypertension.

**Figure 1:**
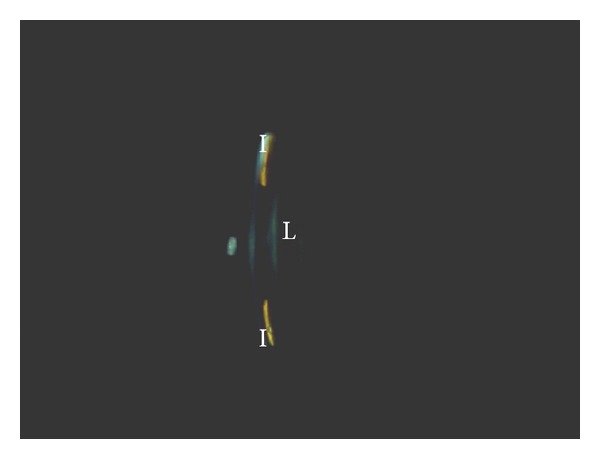
Photo slit camera image of the anterior segment of the left eye. The anterior surface of the lens (L) is in touch with the posterior surface of the iris (I), causing pupillary block thus causing intraocular pressure raise.

On examination at referral, best-corrected visual acuity was 6/10 in his right eye and 6/12 in his left eye. Refraction was −2.5 spherical in the right eye and −3.25 spherical in the left eye. The intraocular pressure was 8 mmHg in his right eye and 26 mmHg in his left eye. He had bilateral neodymium: YAG laser peripheral iridectomies. Both eyes had a shallow anterior chamber. In the left eye, the lens was located slightly forward causing a pupillary block, and the anterior chamber angle was very narrow on indentation gonioscopy (Figure 1). C/D ratio was 0.3 in the right eye and 0.4 in the left eye. Retina was normal in both eyes. Full-threshold visual fields were normal in both eyes. The sagittal lens diameter was 5.35 mm in the right eye and 5.70 mm in the left eye (the mean sagittal lenticular diameter in a young adult's eye is 3.7 mm ± 0.26 SD and in spherophakia 4.5 to 4.9 mm) [3].

**Figure 2:**
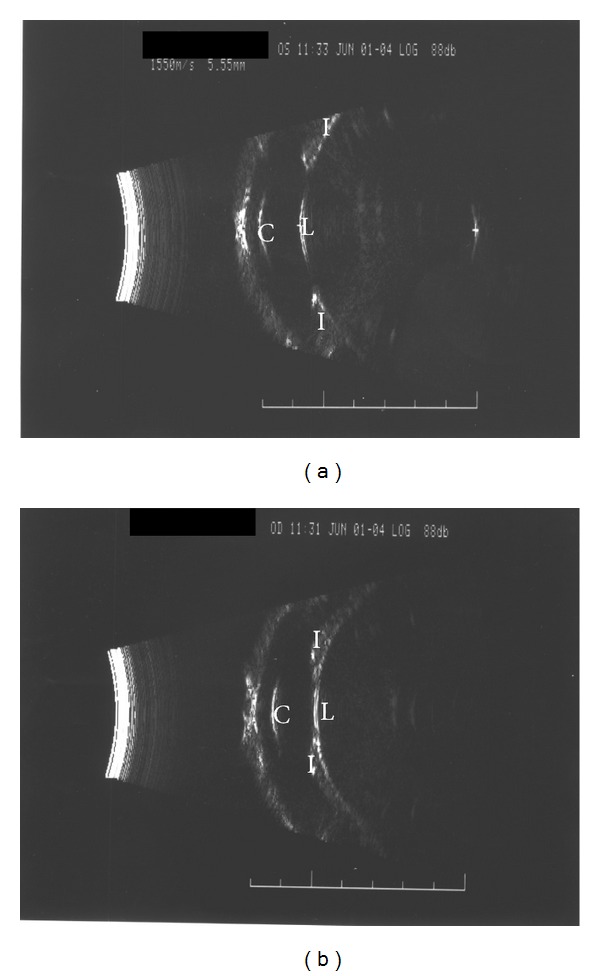
Ultrasound biomicroscopy image of the right eye (a) and the left eye (b) showing the forward luxation of the lens (L) and its touch with the iris (I), thus forming pupillary block, pushing the iris towards the cornea (C), and causing a shallow anterior chamber. Notice the increased anteroposterior diameter of the lens (L).

Ultrasonographic A-scan biometry recorded axial length of 22.2 mm in the right eye and 22.15 mm in the left eye. Ultrasound biomicroscopy with a 50 mHz probe showed a spherophakia lens in both eyes and pupillary block in the left eye due to forward subluxation of the lens (Figure 2). On the basis of these findings, a diagnosis of spherophakia in both eyes was made. In light of the diagnosis of spherophakia the patients' medical history and examination were reviewed. The patient had no history of cardiovascular diseases or skeletal problems and had high intellect. The patient's height was 173 cm with normal skeletal proportions. There were no features of Marfan's syndrome, the Weill-Marchesani syndrome, or homocystinuria.

Chronic pupil block was believed to be responsible for the uncontrolled glaucoma in the left eye. At this stage, we performed lens extraction in the left eye by phacoemulsification, and we implanted a foldable 3-piece acrylic intraocular lens. The postoperative course was routine. The intraocular pressure was controlled with no antiglaucoma drugs, the anterior chamber depth was normal, and the final uncorrected visual acuity was 6/9.

The patient had the same surgical procedure in his right eye with an excellent outcome.

## 3. Comment

Isolated spherophakia, with no association with systemic diseases, is a rare condition [1, 2]. The triad of angle-closure glaucoma, shallow anterior chamber, and myopia should alert the clinician to the possible diagnosis of spherophakia [1]. Myopia is usually high and develops in the second decade [2]. Myopia is mainly lenticular in origin, resulting from the increased lenticular curvature and forward placement of the lens. Axial myopia may also occur; however, axial lengths are usually normal [1]. In our patient, spherophakia occurred as an isolated condition in eyes with moderate myopia and normal axial lengths. 

**Figure 3:**
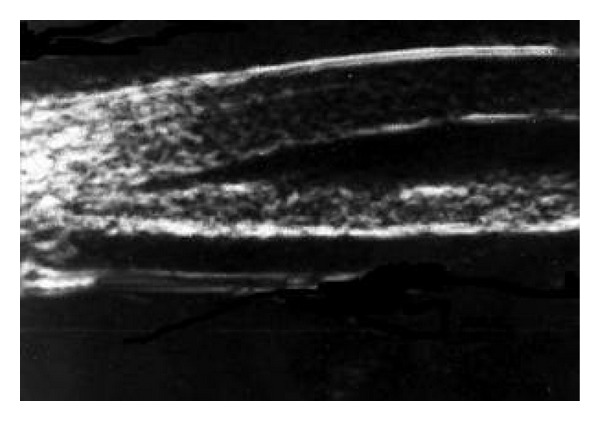
Ultrasound biomicroscopy image of left eye showing hyperplasia of ciliary body.

Previous ultrasonography studies in spherophakia report features similar to our findings in this case [3]. Marchesani suggested that the mechanism of spherophakia is hyperplasia of the ciliary body, resulting in maximum accommodation and lenticular myopia. The hypoplastic ciliary body found with ultrasound was unexpected when first described, and therefore, it is thought now that the fetal lens in spherophakia, which is physiologically spherical naturally, has never been subjected to the force of properly acting ciliary body and zonular fibers [2, 3]. In our patient, there was evidence of hyperplasia of the ciliary body on UBM examination (Figure 3). 

Glaucoma is mainly reported in the literature when spherophakia is associated with the Weill-Marchesani syndrome [2–4]. Glaucoma in isolated and familial spherophakia is less common [1]. Angle-closure glaucoma occurs in spherophakia from a pupillary block mechanism caused by dislocation of the lens and its forward movement, sometime beyond the pupil into the anterior chamber, depending on zonular fibers integrity [4]. 

When the zonular fibers are intact, the lens moves forward and the anterior surface of the lens comes into contact with the posterior surface of the iris and creates pupillary block. The zonular fibers are typically long in the Weill-Marchesani syndrome, and loosening of the zonular fibers allows the lens to move forward, producing lens-iris contact [4].

Chronic intraocular pressure elevation in spherophakia can occur by a variety of mechanisms. Unrelieved pupil block can lead to peripheral anterior synechies and irreversible trabecular damage. Chronic pupillary block and posterior synechies can occur as well as crowding of the trabeculae [3].

Pupillary block is exacerbated with miotics and relieved by mydriatics. Cycloplegic agents relax the ciliary muscle, tighten zonular fibers support, and cause posterior lens movement [4]. 

Peripheral iridectomy has been suggested as a mean to relieve pupil block; however, the rate of surgical complications is high. Vitreous loss occurs frequently as the vitreous face is unprotected by the lens periphery and zonules. Peripheral iridectomies are hardly done any more, and the usual preferred way of treatment is to perform iridotomies by Nd:YAG laser. An Nd:YAG laser peripheral iridotomy is a safer initial procedure and if unsuccessful can be followed by a surgical peripheral iridectomy [5].

In our patient papillary block occurred though the patient had prior yag laser iridotomies most probably due to the iridotomies being not potent. Some of the iridotomies were not peripheral and might be blocked by the anterior movement of the lens.

The role of lens extraction in the management of spherophakia glaucoma has not been established. Surgical removal of the lens may be required to control glaucoma; however, there is a high risk of complications, especially vitreous loss [5]. In spherophakia, the combination of small capsular bag with a relatively high equatorial diameter and zonular fibers instability predisposes to intraoperative and postoperative complications. Shallow anterior chamber, peripheral anterior synechias, posterior synechias, and elevated intraocular pressure may cause surgical difficulties too. In our patient, a regular cataract extraction was done by phacoemulsification through a 1.8 mm opening, and an aspheric foldable posterior chamber intraocular lens (6 mm optic diameter and 12 mm hepatic diameter) was inserted with no difficulty or complications.

In our case, lens extraction was beneficial for the patient who has now normal intraocular pressure without any need for further treatment. The intra- and postoperative courses were uneventful.

This case demonstrates the presentation and pathogenesis of glaucoma in spherophakia and raises several issues about the management of glaucoma in spherophakia. Though lensectomy in spherophakia can be surgically and postoperatively challenging, we suggest considering it as a possible treatment in these cases.
